# Hukou-based rural–urban disparities in maternal health service utilization and delivery modes in two Chinese cities in Guangdong Province

**DOI:** 10.1186/s12939-021-01485-4

**Published:** 2021-06-22

**Authors:** Menghan Shen, Yushan Wu, Xin Xiang

**Affiliations:** 1 Center for Chinese Public Administration Research, School of Government, Sun Yat-sen University, Guangzhou, China; 2 The Jockey Club School of Public Health and Primary Care, Chinese University of Hong Kong, Shatin, N. T. HKSAR China; 3 Graduate School of Education, Harvard University, 14 Appian Way, MA 02139 Cambridge, USA

**Keywords:** Maternal health, Healthcare access, Rural-urban inequality, China

## Abstract

**Background:**

Most existing research on rural–urban health inequalities focuses on disparities in service access and health outcomes based on region. This paper examines rural–urban disparities in maternal healthcare utilization and delivery modes based on household registration (hukou) status to understand the role of state institutions in producing healthcare disparities in China.

**Methods:**

Utilizing administrative data from the Public Maternal Health Insurance scheme, we analyzed 54,733 live births in City A (2015–2019) and 25,849 live births in City B (2018–2019) in Guangdong Province in China. We constructed regression models using hukou status (rural versus urban) as the explanatory variable.

**Results:**

While there is no statistically significant difference in rural and urban mothers’ probability of obtaining the minimum recommended number of prenatal care checkups in City A (OR = 0.990 [0.950, 1.032]), mothers with rural hukou status have a lower probability of obtaining the minimum recommended number of visits in City B than their counterparts with urban hukou (OR = 0.781 [0.740, 0.825]). The probability of delivering in tertiary hospital is lower among mothers with rural hukou than among those with urban hukou in both cities (City A: OR = 0.734 [0.701, 0.769]; City B: OR = 0.336 [0.319, 0.354]). Mothers with rural hukou are more likely to have a Cesarean section than those with urban hukou in both cities (City A: OR = 1.065 [1.027, 1.104]; City B: OR = 1.127 [1.069, 1.189]). Compared with mothers with urban hukou, mothers with rural hukou incurred 4 % (95 % CI [-0.046, -0.033]) and 9.4 % (95 % CI [-0.120, -0.068]) less in total medical costs for those who delivered via Cesarean section and 7.8 % (95 % CI [-0.085, -0.071]) and 19.9 % (95 % CI [-0.221, -0.177]) less for those who delivered via natural delivery in City A and City B, respectively.

**Conclusions:**

Rural hukou status is associated with younger age, no difference or lower probability of having a minimum number of prenatal checkups, higher likelihood of delivering in nontertiary hospitals, increased Cesarean delivery rates, and lower medical cost for delivery in these two Chinese cities. Evaluating how hukou status influences maternal healthcare in Chinese cities is important for devising targeted public policies to promote more equitable maternal health services.

## Introduction

The literature on rural–urban differences in maternal health has generally focused on inequalities created by geographic differences. For example, studies have found that mothers living in rural communities (particularly those in most developing countries) tend to have more restricted access to healthcare (including prenatal checkups, attendance by medical professionals, and Cesarean section) [1–4] and higher rates of adverse obstetric symptoms and maternal mortality than their counterparts living in urban communities [5–10]. These disparities are usually attributed to the lower socioeconomic status of rural residents and the lower density of healthcare providers in rural communities [1, 11–14].

However, little is known about how social institutions affect inequalities in maternal healthcare utilization in China [15]. Rural–urban disparities in healthcare have an additional and critical institutional dimension due to China’s unique household registration (hukou) system. Hukou has been considered the most important determinant of differential privileges and welfare in China [16]. First established in the 1950 s, the hukou system served as an institutional basis for controlling migration by restricting individuals’ entitlement to social rights, such as public schooling [17], job opportunities [16], housing [16], and medical insurance [18], based on their place and type of hukou registration, with all residents classified into agricultural (rural) or nonagricultural/residential (urban) categories [19]. Though direct restrictions on domestic migration gradually loosened in the 1980 and 1990 s, changing one’s hukou registration remained difficult [20]. Therefore, migrants of rural origin usually retain their rural hukou status long after they have settled in urban communities, unable to obtain full social rights as urban residents. The majority also face institutional discrimination, social stigma, and marginalization in urban areas [21, 22]. As a result of differential access to opportunities and resources, individuals with rural hukou generally have less education and fewer economic resources than those with urban hukou [23–25].

Studies have consistently reported hukou-based rural–urban disparities in access to healthcare and health outcomes across the life course [15, 26–29], demonstrating the distinct role of state institutions in producing rural–urban inequalities in China. Despite residing in urban communities, migrants retaining rural hukou status are less likely to utilize healthcare services and have worse health outcomes than urban residents and migrants with urban hukou status [15, 24, 26, 30, 31]. Researchers usually attribute these disparities in access and outcomes to rural-hukou holders’ lower socioeconomic status and the hukou-based disparities in insurance coverage [23, 25, 26, 32–35]. Rural-hukou holders tend to have less formal education and fewer economic resources, contributing to their more limited knowledge about healthcare and ability to pay for out-of-pocket costs [23–25]. In the urban context, rural-hukou holders tend to experience more stringent work conditions and schedules that prevent them from seeking care [23, 25]. Furthermore, residents with rural hukou status and those with urban hukou status are eligible for different basic health insurance programs at the place of their hukou registration, with the rural program providing more limited coverage [26, 35, 36]. Regardless of their hukou status, individuals working in urban communities are eligible for the Urban Employee Basic Medical Insurance (UEBMI) program, which generally provides better coverage than the urban and rural residency-based basic insurance programs. Nonetheless, workers with rural hukou status are less likely to obtain formal labor contracts and enroll in the UEBMI program at their place of work and residence than their counterparts with urban hukou [23, 25, 32, 37, 38]. To address these disparities, the Chinese state began to gradually integrate these basic health insurance programs in the mid-2010 s [36, 39]. However, the integration process is far from complete and the efficacy and impact of these efforts on hukou-based rural–urban disparities remain to be evaluated.

Despite the importance of rural–urban hukou status in determining access to healthcare and health outcomes, few studies have systematically examined hukou-based rural–urban disparities in maternal health. The limited existing research on this topic mainly focuses on describing and explaining the disadvantages experienced by rural-to-urban migrant mothers compared with urban mothers [30, 31, 40–42], particularly in large cities with high concentrations of migrants. Though the comparison between migrant mothers and urban mothers has important theoretical and practical implications, it confounds the effects of migration and the effects of the hukou institution.

Because of the large share of rural hukou population in China and the extensive impact of maternal healthcare access on life-long outcomes [43, 44], assessment of rural–urban hukou-based disparities in maternal health is indispensable when working towards a more efficient and more equitable healthcare system. To fill the literature gap, in this study, we utilize administrative data from the Public Maternity Health Insurance (PMHI) scheme to examine hukou-based rural–urban disparities in maternal healthcare utilization among urban formal employees in two midsize cities in the Pearl River Delta. We focus on three important indicators that can be extracted from this administrative dataset: whether the mother obtained the minimum recommended number of prenatal care checkups (eight or more), mode of delivery (Cesarean section versus natural delivery), the level of the hospital (tertiary hospital or not), and fees incurred for delivery. Like the UEBMI program, the PMHI program is a mandatory insurance scheme for urban employees with formal labor contracts regardless of gender or hukou status. Informal workers and unemployed people can also join voluntarily. It provides reimbursements for prenatal care and deliver for enrolled employees and their unemployed spouses. By using PMHI data, we are able to exclude the impact of hukou-based differences in insurance coverage and provide a lower-bound estimate of hukou-based rural–urban disparities in maternal healthcare in urban China. In other words, we ask whether hukou-based rural–urban disparities in maternal healthcare persist even among those enrolled in the same public insurance scheme. The answer to this question is critical for evaluating the importance of hukou status in influencing maternal healthcare in Chinese cities and for devising targeted public policies to promote more equitable maternal healthcare services.

## Methods

### Data

We utilized administrative data from the PMHI scheme in two coastal cities in China. For anonymity, we call them City A and City B. Both are midsize coastal cities in the Pearl River Delta of Guangdong Province. As demonstrated in Table 1, City A is typical of midsize coastal cities in Guangdong in its demographic and socioeconomic characteristics. City B, in contrast, is one of the most prosperous midsize cities in the Pearl River Delta, with faster population growth, a higher percentage of urban residents, and a higher GDP per capita than average. These two cities were selected for this study because of data availability. We utilized data from two cities to see whether cities that differ in their population and economic development would exhibit similar trends in rural–urban hukou-based disparities on indicators of maternal healthcare service utilization.

**Table 1:** Basic Characteristics of City A, City B, and the Average City in Guangdong (2019)

	City A	City B	Guangdong Avg
Residents	4–5 million	2–3 million	5.5 million*
Annual population growth	0–1 %	6–8 %	1.5 %
Percentage of residents living in urban neighborhoods	60–70 %	80–90 %	71.4 %
Per-capita GDP (yuan)	65,000–70,000	170,000–180,000	94,172
Annual per capita GDP growth	3–4 %	6–7 %	4.5 %

Note: All data are based on the annual statistical reports of the prefecture and provincial governments in 2019. To ensure the anonymity of these two cities as required by our data use agreement, we give them pseudonyms and provide ranges rather than exact figures for their demographic and socioeconomic characteristics.

^*^This is the average population for the 21 prefecture-level cities in Guangdong.

We obtained access to and analyzed 20 % of the randomly selected PMHI data from January 2015 to December 2019 for City A and 100 % of the PMHI data from October 2018 to December 2019 for City B. The PMHI data contain two automatically generated datasets that are pseudonymized but linked at the individual level: (1) an enrollment records dataset, which includes individuals’ enrollment status and demographic characteristics, such as hukou status, age, and gender; and (2) a claim records dataset, which includes key information for each insurance claim, such as date of visit, provider facility name, provider type and level, district of residence within the city, diagnosis code (International Classification of Diseases [ICD-10] code), delivery mode (natural delivery or Cesarean section), and total medical expenses.

### Variables

The primary independent variable is the PMHI enrollee’s hukou status (rural versus urban), which we derive from the enrollment records dataset. The primary outcome variables of interest come from the claim records dataset and include maternal age, whether mothers had at least eight prenatal care checkups (the minimum number recommended by the World Health Organization) [45], whether the hospital where the woman delivers is a tertiary hospital, mode of delivery (natural delivery versus Cesarean section), and total fees for delivery. Total medical cost is the sum of treatment, operation, drug, and other expenses. Because prices for medical services are set by China’s Ministry of Health, variations in total fees generally reflect differences in the utilization of medical services [46].

### Statistical analysis

We analyzed data for 54,733 live births in City A (2015–2019) and 25,849 live births in City B (2018–2019) to compare birth-related outcomes of mothers with urban hukou status versus rural hukou status. The number of births in our dataset corresponds to roughly 20 % of all births in City A and 100 % of all births in City B during this period. We present means and standard deviations for age and conduct a *t*-test to compare means between the two groups. We present medians and standard deviations for spending and conduct a Mann-Whitney test to compare medians between the two groups. We present percentages and the number of observations for whether a Cesarean section was performed at delivery and whether the delivery took place at a tertiary hospital and conduct χ2 tests to compare means between the two groups. We used mothers with urban hukou status as the reference group.

We used an ordinary least squares model to analyze outcomes related to age and total medical spending. Because total costs were skewed to the right, we transformed costs into their natural logarithm and replaced zeroes with the minimum value minus 0.01. We used logistic models to analyze outcomes for whether the delivery took place at a tertiary hospital and whether a Cesarean section was performed. We controlled for year and month fixed effects for our analysis on age. In our analyses for other outcomes, we included maternal age as a control variable. We used robust standard errors to correct for heteroskedasticity. All statistical analyses were conducted in STATA 16. We obtained ethical approval (SBRE-19-770) from the Chinese University of Hong Kong.

## Results

As shown in Table 2, the average maternal age was 29.47 (*SD* = 4.20) years for mothers with rural hukou status and 31.27 (*SD* = 4.73) years for mothers with urban hukou status in City (A) The average maternal age was 29.88 (*SD* = 4.06) for mothers with rural hukou status and 31.97 (*SD* = 4.54) for mothers with urban hukou status in City (B) Mothers with rural hukou status were, on average, about two years younger than those with urban hukou status in both cities (Coef: -1.912; 95 % CI [-1.989, -1.835]) (Table 3).

**Table 2:** Characteristics, Healthcare Service Utilization, and Delivery Modes of Mothers with Rural and Urban Hukou Status in City A and City B

	City A				City B			
	Rural	Urban	T-Value	*P*	Rural	Urban	T-Value	*P*
Age	29.47	31.27	46.44	< .001	29.88	31.97	38.77	< .001
	(4.20)	(4.73)			(4.06)	(4.54)		
	Rural	Urban	Chi-square	*P*	Rural	Urban	Chi-square	*P*
Reached minimum recommended number of prenatal care visits	18,588	23,407	0.34	.560	7,732	9,130	45.43	< .001
	76.84%	76.63%			62.08%	68.18%		
Delivery at tertiary hospital	3,705	6,051	186.11	< .001	4,802	8,948	2100.00	< .001
	15.32%	19.81%			38.55%	66.81%		
Delivery via Cesarean section	8,994	12,275	51.33	< .001	4,714	5,445	21.37	< .001
	37.18%	40.19%			37.85%	40.66%		
	Rural	Urban	Z-statistics	*P*	Rural	Urban	Z-statistics	*P*
Total medical cost								
Delivery via Cesarean section	6197.37	6424.16	13.61	< .001	8572.31	9571.51	20.37	< .001
	(2035.09)	(2532.35)			(4179.88)	(3992.57)		
Natural delivery	3372.83	3574.56	21.10	< .001	4687.44	6055.16	33.66	< .001
	(2857.45)	(2020.54)			(2331.49)	(2788.77)		
N	24,189	30,544			12,456	13,393		

Note. We present means and standard deviations for age and conducted a t-test to compare means between the two groups. We present medians and standard deviations for spending and conducted a Mann-Whitney test to compare medians between the two groups. We present percentages and number of observations for whether the delivery took place at a tertiary hospital and whether a Cesarean section was performed at delivery and conducted χ^2^ tests to compare means between the two groups. We used mothers with urban hukou status as the reference group.

^*^p < .1, ^**^p < .05, ^***^p < .001

**Table 3:** Maternal Hukou Status and Age

	City A	City B
	Coef	
Age	-1.912***	-2.059***
	[-1.989, -1.835]	[-2.164, -1.955]
R-squared	0.07	0.07
*N*	54,733	25,849

Note. We used an ordinary least squares model to analyze outcomes on age and log of total medical spending. We used logistic models to analyze the outcomes on whether the mother had obtained the minimum recommended number of prenatal care checkups, whether a Cesarean section was conducted, and whether the delivery took place at a tertiary hospital. For all of the regressions, we included maternal age and dummy variables for year and month. We used mothers with urban hukou status as the reference group. 95% confidence intervals are shown in brackets.

^*^p < .1, ^**^p < .05, ^***^p < .001

In City A, the probability of having the minimum recommended number of prenatal care visits was 76.84 % for mothers with rural hukou status and 76.63 % for mothers with urban hukou status. As shown in Table 4, there was no statistically significant difference between the two groups (*OR* = 0.990 [0.950, 1.032]). In City B, the probability of obtaining the minimum recommended number of prenatal care visits was lower for mothers with rural hukou status than for mothers with urban hukou status (62.08 % versus 68.18 %). After controlling for age and time fixed effects, mothers with rural hukou status continued to have a lower probability of obtaining the minimum recommended number of prenatal care visits than their counterparts with urban hukou status (*OR* = 0.781 [0.740, 0.825]).

The probability of delivering in a tertiary hospital was 15.32 and 19.81 % for rural hukou and urban hukou mothers in City A and 38.55 and 66.81 % in City B, respectively. For both cities, the probability of delivering in a tertiary hospital was lower among rural hukou mothers than among their counterparts with urban hukou status (City A: *OR* = 0.734 [0.701, 0.769]; City B: *OR* = 0.336 [0.319, 0.354]) (Table 4).

The probability of delivering via Cesarean section was 37.18 and 40.19 % for rural hukou and urban hukou mothers in City A and 37.85 and 40.66 % in City B, respectively. However, after controlling for age, rural mothers were more likely to have a Cesarean section than were those with urban hukou (City A: *OR* = 1.065 [1.027, 1.104]; City B: *OR* = 1.127 [1.069, 1.189]) (Table 4).

For those who delivered via Cesarean section, the average total medical cost was 6197.37 yuan for mothers with rural hukou status and 6424.16 yuan for mothers with urban hukou status in City A and 8572.31 yuan for mothers with rural hukou status and 9571.51 yuan for mothers with urban hukou status in City B. For those who delivered naturally, the average total medical cost was 3372.83 yuan for mothers with rural hukou status and 3574.56 yuan for mothers with urban hukou status in City A. In City B, the average total medical cost was 4687.44 yuan for mothers with rural hukou status and 6055.16 yuan for mothers with urban hukou status. As shown in Table 4, our regression analysis suggests that compared with mothers with urban hukou status, mothers with rural hukou status incurred 4 % (95 % CI [-0.046, -0.033]) and 9.4 % (95 % CI [-0.120, -0.068]) less in total medical costs when they delivered via Cesarean section and 7.8 % (95 % CI [-0.085, -0.071]) and 19.9 % (95 % CI [-0.221, -0.177]) less when they delivered via natural delivery in City A and City B, respectively.

**Table 4:** Maternal Hukou Status and Birth-Related Outcomes

	City A	City B
	Odds Ratio	
Reached minimum recommended number of prenatal care visits	0.990	0.781***
	[0.950, 1.032]	[0.740, 0.825]
*N*	54,724	25,842
Delivery at tertiary hospital	0.734***	0.336***
	[0.701, 0.769]	[0.319, 0.354]
*N*	54,729	25,839
Delivery via Cesarean section	1.065***	1.127***
	[1.027, 1.104]	[1.069, 1.189]
*N*	54,729	25,844
	Coef	
Total medical cost		
Cesarean section	-0.040***	-0.094***
	[-0.046, -0.033]	[-0.120, -0.068]
R-squared	0.09	0.01
*N*	21,269	10,159
Natural delivery	-0.078***	-0.199***
	[-0.085, -0.071]	[-0.221, -0.177]
R-squared	0.13	0.03
*N*	33,464	15,690

Note. We used logistic models to analyze whether Cesarean section was conducted and whether the delivery took place at a tertiary hospital. We used an ordinary least squares model to analyze outcomes on the log of total medical spending. For all of the regressions, we included maternal age and year and month dummies as control variables. We used urban mothers as the reference group. 95% confidence intervals are shown in brackets.

^*^p < .1, ^**^p < .05, ^***^ *p* < .001

## Discussion

In both cities in our study, rural hukou status is associated with younger maternal age, lower probability of having the minimum recommended number of prenatal care checkups, reduced likelihood of delivering at a tertiary hospital, higher likelihood of delivering via Cesarean section, and lower fees for delivery. While the two cities differ in their population and economic development, they exhibit similar trends in hukou-based rural–urban disparities on most of the indicators of maternal healthcare service utilization we examined. These findings confirm the existence of hukou-based rural–urban disparities in maternal healthcare even among urban formal employees enrolled in the same public insurance scheme. Given that all of our participants are enrolled in the same insurance scheme, the hukou-based rural–urban disparities in prenatal care checkups in City B are likely a result of hukou-based differences in socioeconomic factors, such as income, inflexible job schedule, and educational level, which have been reported as important determinants of the use of prenatal care and maternal healthcare services both in China and internationally [47–49].

Adequate maternal care is a precondition for identifying and mitigating potential risk factors for adverse pregnancy outcomes and reducing pregnancy-related mortality and morbidity [50]. Having eight or more prenatal care checkups is associated with a lower probability of mortality and health complications for mothers and children, compared with having fewer prenatal care checkups [51, 52]. Our result is consistent with previous literature showing that residents with rural hukou status in the city underutilize healthcare compared with their urban hukou counterparts in both outpatient and inpatient settings, possibly because of budget and time constraints [26].

Notably, as the first study examining rural-urban hukou-based disparities in delivery mode, this paper reports a novel and surprising finding that Cesarean delivery rates were higher among mothers with rural hukou status after controlling for maternal age. While Cesarean deliveries can save lives, they are also associated with short- and long-term complications and higher costs for both families and healthcare systems [43, 44]. In China, the high percentage of medically unnecessary Cesarean deliveries has been a cause of concern due to widespread false beliefs that Cesarean sections are safer than natural delivery [53] and moral hazard problems on the supply side [54]. In most developing countries, Cesarean delivery rates are lower in rural communities than in urban ones [10, 55, 56]. Studies examining delivery modes in China have consistently reported similar patterns in the past two decades [47, 48, 57], though more recent data show an increase in Cesarean delivery rates among rural residents [58, 59], possibly due to improvements in their financial means [60], increased rates of medical insurance and financial protection [61], and increased availability and improvement of healthcare services in rural regions [59]. Nevertheless, all of these studies still found higher Cesarean section rates in urban areas [58, 59, 62]. Given these prior findings, one would expect that mothers with rural hukou status would be less likely to have Cesarean deliveries than their counterparts with urban hukou status, if there are any hukou-based disparities at all.

There are several possible explanations for our surprising finding on delivery mode. First, with aggregated data, most existing studies did not control for age in examining the relationship between rurality and Cesarean delivery rates [58, 59]. The higher maternal age in urban communities may partly account for their Cesarean delivery rates. Second, existing studies compared mothers living in rural communities with those in urban communities, who likely had different types of health insurance and different access to healthcare facilities. Previous studies considering moral hazard problems on the supplier side have reported that having public health insurance and living in a region with access to hospitals with the capacity to perform Cesarean surgeries are associated with higher risks of Cesarean delivery [57, 61]. Since the women in our dataset are all covered by the PMHI scheme and reside in the same city, women with rural hukou status may have elevated Cesarean delivery rates because they are exposed to a similar set of risk factors as urban mothers but perhaps remain less aware of the risks of Cesarean section. Third, studies comparing differences in delivery modes by rural–urban residence and socioeconomic status have mostly used data from before 2014 [47, 48, 57]. Our results may reflect new patterns in Cesarean deliveries in China: Cesarean deliveries may have become so commonly available and overused that socioeconomically disadvantaged groups are now more likely to be taken advantage of by going through unnecessary Cesarean deliveries.

Despite efforts to improve the primary care system in China, tertiary hospitals are generally considered higher-quality than nontertiary hospitals [63]. Cost and distance likely contribute to the lower likelihood of delivering at tertiary hospitals among mothers with rural hukou status. First, there is usually a high level of residential segregation by hukou status. Whether they are local rural mothers residing in the suburbs or rural-to-urban migrant mothers living in cramped migrant neighborhoods, mothers with rural hukou status tend to live further away from the central urban districts where tertiary hospitals are generally located [64]. Second, tertiary hospitals generally cost much more than nontertiary hospitals for two reasons (in our case, 900–3000 yuan more on average). On one hand, China’s Ministry of Health sets a higher price for services utilized in tertiary hospitals [46]. On the other hand, tertiary hospitals have more advanced technologies [65], and the associated checkups and treatments cost more. With more constrained budgets [66], mothers with rural hukou status may be less likely to choose tertiary hospitals.

Mothers with rural hukou status spent less on inpatient care regardless of their delivery method than their counterparts with urban hukou status. The hukou-based disparities in fees are likely the result of hukou-based differences in mothers’ choice of tertiary versus nontertiary hospitals and inpatient care packages (usually based on the quality of equipment used). Mothers with rural hukou status may also have been more likely to express cost-related concerns to their physicians, which might lead to physicians prioritizing cost reduction when prescribing medications and services [67]. However, because there is no evidence to suggest that higher medical spending is associated with better quality of care in developed countries [68], the implications of differentials in spending by hukou status are less clear. More research should be done in developing countries like China to understand the relationship between medical spending and healthcare outcomes.

This study has unique strengths that enable it to provide fresh insights into rural–urban disparities in maternal healthcare. First, to the best of our knowledge, it is the first study to evaluate hukou-based rural–urban disparities in maternal healthcare service utilization in China and it uses the most recent data. Second, our use of individual-level datasets allows us to control for maternal age when evaluating the impact of hukou status on maternal healthcare service utilization and delivery mode, an important factor that most existing studies did not account for. Last, our dataset allows us to examine outcomes related to healthcare utilization as well as obstetric outcomes.

The main limitation of this study is the selection bias inherent in the PMHI datasets. Because participation in the PMHI scheme is only mandatory for those working in the formal sectors and voluntary for those who are self-employed, our datasets do not include residents who themselves as well as their spouses are unemployed or are employed in informal sectors and opted for nonparticipation. As mothers with rural hukou status are more likely to be unemployed or employed in informal sectors [69], they are more likely not to have PMHI coverage. Thus, we might find a larger disparity between women with rural and urban hukou status if we took into account women who are not enrolled in the PMHI. In other words, by restricting the sample to women with PMHI coverage, we are underestimating the size of hukou-based rural–urban disparities. However, understanding this difference conditional on having the same type of health insurance is important in light of the recent health insurance integration efforts.

Another limitation is that we lack data on key indicators of maternal and infant health, such as maternal mortality and neonatal mortality. Moreover, we do not have sufficient information to determine whether prenatal care began in the first trimester, what quality of prenatal care was offered, whether the costs associated with delivery were appropriate or sufficient, and whether delivery via Cesarean section was necessary. However, because close to 40 % of the births in our dataset were Cesarean sections, which is substantially higher than the 10–15 % rate recommended in most maternal health guidelines [70], it seems likely that the Cesarean method was overused.

It is also worth noting that while most of the differences in outcomes by mothers’ hukou status are similar in these two cities, whether the mother obtains the minimum recommended number of prenatal care checkups differs. Notably, City A has a higher level of women obtaining the minimum recommended number of prenatal care visits, with no difference between mothers with rural and urban hukou status. More research is needed to understand the difference between cities.

Finally, we lack sufficient data to determine the mechanisms behind hukou-based rural–urban disparities in maternal healthcare utilization and delivery modes. The insurance administrative datasets we used do not contain reliable information about mothers’ previous hukou status, educational level, income level, occupation status and previous health conditions. Neither do they contain information about maternal death and complications, which are also key indicators of maternal health outcomes. Future research utilizing survey data with more comprehensive individual-level information may further explore the mechanisms underlying the hukou-based disparities that we report.

The disparities in prenatal checkups, choice of hospital, and medical spending for delivery between rural and urban hukou holders in the same city suggest the importance of hukou status in determining maternal healthcare utilization, even when women have the same type of public health insurance and reside in the same city. In other words, policymakers need to be aware that consolidating health insurance may not eliminate disparities in healthcare utilization. Strategies to improve outcomes for mothers with rural hukou status should include targeted efforts to increase utilization of prenatal care among mothers with rural hukou. For example, there could be targeted campaigns to increase awareness of prenatal care services and additional subsidies for prenatal care for mothers with rural hukou. Because of the major variations in the cesarean rates and trends, understanding the types of women with elevated Cesarean delivery rates can be particularly informative in reducing the rate of medically unnecessary Cesarean sections, which rose between 2017 and 2018 after a temporary decrease of caesarean birth rate appeared in 2016 [58, 59]. Targeted health education and publicity can also reduce unnecessary Cesarean sections among women with rural hukou status by instilling confidence in the safety of natural deliveries and promoting understanding of maternal and neonatal safety. Furthermore, supply-side strategies that focus on payment mechanism and healthcare provider practice should be continually enforced to reduce non-essential cesareans. Finally, our findings suggest the persistence of hukou-based disparities in healthcare utilization and outcomes. Reform of the hukou system needs to be deepened and accelerated to reduce hukou-based inequities in health in contemporary China.

## Conclusion

Using data from the PMHI program in two cities, we found that women with rural hukou status were younger when they gave birth, made fewer prenatal care visits, incurred lower fees for delivery, and were more likely to deliver via Cesarean section than their counterparts with urban hukou status. Accessibility of prenatal care and awareness of the benefits of natural delivery should be further promoted for this group of women. Understanding the types of women with elevated Cesarean section rates can inform a targeted approach to reducing Cesarean section rates overall.

## Data Availability

The datasets generated and/or analyzed in the current study are not publicly available but are available from the first author on reasonable request.
